# The Emergence of Planetary Pediatrics

**DOI:** 10.3390/children8100939

**Published:** 2021-10-19

**Authors:** Charles Oberg

**Affiliations:** Global Pediatric Program, Department of Pediatrics, University of Minnesota, Minneapolis, MN 55415, USA; oberg001@umn.edu

**Keywords:** planetary pediatrics, climate change, environment, social justice, violence against children, child migration, global health

## Abstract

Pediatrics has witnessed an evolution from primary care, family-centered care, community pediatrics, social pediatrics and global pediatrics, which has shifted our attention beyond the clinic setting to an appreciation of children in their lived environment. We are witnessing the emergence of planetary pediatrics that further broadens the focus of children’s health to include the continued importance of clinical care, but also the impacts of climate change, environmental degradation, child migration, unrelenting war and conflict, social injustice, pandemics and violence against children. If we do not acknowledge the present and ever-increasing adverse planetary changes of what children are experiencing now and in the future, we will have failed to adequately protect them from impending catastrophes. The hope of pediatrics for the future is to improve the health and well-being of all children. This hope remains as relevant today as it was for our predecessors and serves as a beacon for the voyage through the remainder of the twenty-first century.

## 1. Introduction

**Figure 1:**
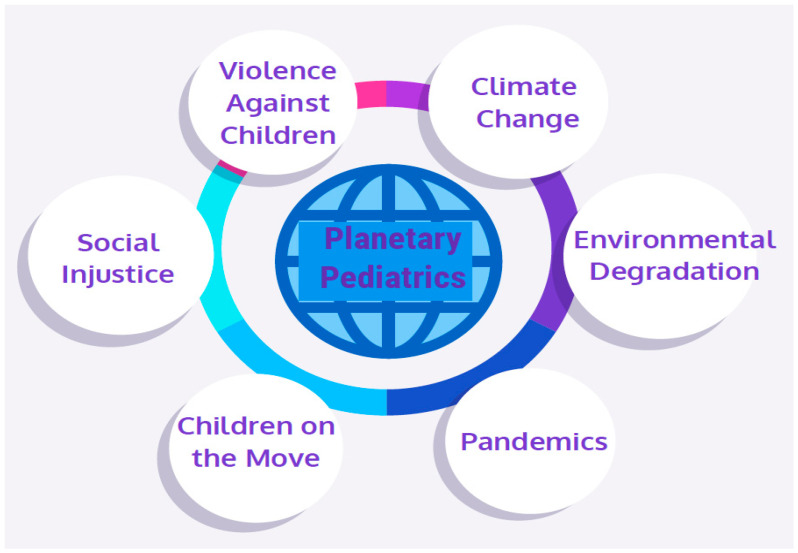
Planetary Pediatric Framework.

Pediatrics over the last one hundred years has exhibited a transformation that has evolved from a focus on the care of the child in the context of general pediatrics and subspecialty care, to a focus on a broader commitment to children. The profession has witnessed the evolution from primary care, family-centered care, community pediatrics, social pediatrics and global pediatrics, which has shifted our attention from the clinic setting, to an appreciation of children in their lived environment. We are witnessing the emergence of planetary pediatrics that broadens this focus further [1]. Global health typically focuses on health improvements for international populations, whereas planetary health, in contrast, examines the health of humanity globally and the health of the earth’s systems on which it depends [2]. Planetary pediatrics continues to embrace the importance of clinical care, but in the context of climate change, environmental degradation, epidemics and pandemics, child migration, unrelenting war and conflict, discrimination and social injustice, and violence against children. Each of these challenges are interrelated, resulting in an urgent need to address them in a concerted manner. Figure 1 depicts the Planetary Pediatric Framework.

## 2. Climate Change

As we move forward through this century, climate change presents the greatest existential threat to humanity. The impact on child survival, optimal growth and development and the capacity to thrive is immeasurable and demands an international effort to reverse the impending catastrophic consequences. UNICEF has determined that there may be no greater threat facing the world’s children, and future generations, than climate change [3].

Children are disproportionately impacted by climate change due to their unique metabolism, physiology and developmental needs. In addition, climate change has a disproportionate impact on selective groups of vulnerable children. These include children on the move, those living in poverty, indigenous children and those with developmental disabilities. The impact is magnified because countries which are the most susceptible to climate change also tend to have a higher proportion of these children [4].

Climate change increases the frequency and intensity of extreme weather events. These events negatively impact children’s ability to access an adequate quality and quantity of food, clean potable water, and appropriate health and social services. Waterborne diseases such as dysentery will increase due to the intensification of flooding. Diarrhea is presently the second leading cause of mortality globally for children under 5 years of age. By 2030, it is projected that climate change impacts will result in 48,000 additional deaths in children under 15 years of age above the projected baseline [5]. The change in climate also impacts the range of vectors, leading to increases in diseases such as malaria, dengue, leptospirosis and leishmaniosis. Finally, climate change and extreme climate events contribute to increased stress, anxiety, depression, and post-traumatic stress disorder (PTSD) [6,7].

## 3. Environmental Degradation

The lack of environmental justice, or the lack of meaningful input and involvement of all people in policy development and the implementation and enforcement of regulations, persists, with those most vulnerable at the greatest risk of experiencing the adverse consequences of such practices [8]. Environmental degradation continues to threaten earth’s sustainability. Deforestation, soil degradation, dependance on plastic and other non-degradable products, the overuse of pesticides and herbicides, and the contamination of global clean water sources all continue despite national and international attempts at mitigation [9]. The exposure to environmental chemicals (such as lead, mercury, dioxin, arsenic and pesticides) and the impact on fetal and child development continues. Environmental exposure disproportionately affects vulnerable communities, with industrial plants, waste facilities, strip mines and other polluters frequently located in close proximity to low-income families with children [10].

## 4. Disease Outbreaks, Epidemics and Pandemics

Since the turn of the millennium, the planet has witnessed a number of disease outbreaks, epidemics that have warranted global attention. These have included the Ebola outbreak in 2014, which began in Western Africa, and the Zika virus epidemic originating in South America in 2018, both of which took on global proportions. Starting in 2019, the COVID-19 epidemic engulfed the world with an estimated 220 million cases and 4.6 million deaths as of September 2021, both considered underestimates of the true magnitude of the pandemic [11]. Although children have not been the overt face of the COVID-19 pandemic, the greatest long-term and unseen impacts may be the effect on the lives of children. Children have experienced profound disruptions in their lives, including school closures, social isolation, loss of family members, worsening conditions of poverty, child labor and child marriage, violence and exploitation. It has had a significant impact on health inequities as well as social isolation, psychosocial well-being and increased exposure to racism, xenophobia, and health inequities [12]. In addition, as the pandemic persists with multiple waves and the Delta variant’s greater transmissibility, children are becoming infected in significantly greater numbers [13].

## 5. Children on the Move—The Global Migration of Children

It has been estimated that more than 50 million children and youth have migrated across borders or been forcibly displaced within their own country since the start of the 21st century [14]. They consist of refugees, asylum seekers, internally displaced persons (IDPs) and economic migrants. This crisis is now of global proportions directly and/or indirectly affecting every one of us. Palestinians remain the largest and longest displaced group of refugees. For decades, the global community seems to have forgotten the children living in the occupied territories of Gaza and the West Bank, as well as those who been dispersed throughout the Middle East in Lebanon, Jordan and Syria.

Every decade has experienced its own diaspora of newly displaced families. This includes, but is not limited to, the exodus from Southeast Asia in the 1970s and 1980s, Somalia in the 1990s and those who have more recently fled the conflicts in South Sudan, Iraq, Afghanistan, Syria, Yemen, Ethiopia and the Rohingya fleeing Myanmar. Children on the move also include millions of children migrating either with their families or as unaccompanied children fleeing poverty, exploitation and violence who are seeking a safer environment and opportunity for the future.

## 6. Discrimination and Social Justice

Discrimination based on race, class, gender and religion continues, with xenophobia on the rise. In the United States, we continue to observe anti-Black, anti-Brown, anti-Asian, anti-Sematic, anti-Muslim and anti-LGBTQ sentiments, at times expressed in violent fashion. Globally, a turn toward nationalistic and autocratic leaders who demonize those who are perceived as different or a threat fuels anger and distrust [15].

## 7. Violence against Children

Violence against children (VAC) in all its forms is an enormous child health issue. This includes child maltreatment as well as those experiencing cyber-bullying, sexual violence and emotional and/or psychological violence [16]. In addition, millions of children live with armed conflict as the norm. Children are caught in the crossfire or are directly targeted by combatants, resulting in injury, illness, disability, psychological trauma and death. Children are recruited and forced into armed groups. Child labor remains ubiquitous globally and is underpinned by poverty and a lack of educational opportunities. Over 168 million children work, with more than half of them performing hazardous tasks. Estimates suggest that 50% of trafficked victims worldwide are children [17]. Exploitive practices involving child marriage, illicit adoption, begging, and organ harvesting are also now included in the VAC domain [18]. Evidence over the past 30 years—from neuroscience, developmental psychology, social sciences and epidemiology—has shown that such violence contributes to social, emotional and cognitive impairments and high-risk behaviors, leading to disease, disability, social problems and premature mortality, with short, medium, long-term and intergenerational consequences.

## 8. Planetary Pediatrics Framework—Interconnection and Interdependence

The major topics discussed are not separate, independent problems that our children will inherit, but are rather interconnected and interdependent. For example, the existential threat of climate change for children is intrinsically interrelated with the other issues identified in the framework. As the climate warms with the resultant droughts, floods and increased frequency and intensity of major weather events, it will exacerbate other emergencies. Deforestation to expand available agricultural land due to shifting seasonal growing patterns will decrease CO_2_ capture and increase soil erosion, thus exacerbating environmental degradation. It also alters the global distribution of pathogens, which results in greater outbreaks of disease. In addition, droughts which may lead to famine frequently contribute to civil unrest and conflict, resulting in greater numbers of refugee children due to forced displacement. Such displacement fuels the social injustices of xenophobia and racism, which further contributes to violence against children.

## 9. Planetary Pediatric Advocacy Strategies for Change

Advocating for change to successfully address the problems that have been outlined will require action that spans individual responsibility to collective action.

### 9.1. Patient Care

A concern for planetary pediatrics has to have relevance in the clinical setting. We can share the impact of climate warming on health, identify violence in all its manifestations and be culturally humble in our approach to care. In addition, pediatricians can help to prevent exposure to environmental hazards by being aware of potential threats and educating about minimizing risk.

### 9.2. Community Engagement

As child health providers, we realize that our families and their children are embedded in the community. Our appreciation and involvement in community affairs helps us to both realize the challenges facing the children we serve, but also provides the opportunity for networks and coalitions that can address such issues on a larger scale.

### 9.3. State and National Advocacy

The American Academy of Pediatrics (AAP), through its state chapters and its national advocacy efforts, provides guidance and training on many of the components of planetary pediatrics. Involvement in state chapters and national committees, sections and councils provides opportunity for the pediatrician to become involved through participation in the Annual Leadership Forum and legislative training. In addition, the Academic Pediatric Association (APA), American Pediatric Society (APS) and the Society for Pediatric Research (SPR) have research, policy and advocacy agendas that are aligned with the planetary pediatric framework themes. Cross-disciplinary collaborations are also essential, such as potential partnerships with the American Psychological Association (APA) and the Society for Research in Child Development (SRCD).

### 9.4. Global Partnerships

Involvement on a global stage may take the form of participating in clinical work overseas throughout training and practice. There are organizations in need of quality pediatric primary and sub-specialty training that include short-term deployment, such as with the AAP-supported Health Volunteers Overseas (HVO) or longer commitments through Doctors without Borders/Médecins Sans Frontières (MSF) and other organizations. In addition, we can develop partnerships with our international colleagues through global professional organizations such as the International Pediatric Association (IPA) or the International Society of Social Pediatrics and Child Care (ISSOP).

## 10. Conclusions

The optimal care of children is the cornerstone of the profession. However, if we do not acknowledge the present and ever-increasing adverse planetary changes of what children are experiencing now and in the future, we will have failed to adequately protect them from impending catastrophes. The hope of pediatrics for the future is to improve the health and well-being of all children. This hope remains as relevant today as it was for our predecessors and serves as a beacon for the voyage through the remainder of twenty-first century.

## Data Availability

Not applicable.
